# Recovery from chronic fatigue syndrome: a reflexive thematic analysis of experiences of people before, during and after treatment

**DOI:** 10.1080/17482631.2025.2595829

**Published:** 2025-12-05

**Authors:** Tom Ingman, Trudie Chalder, Vanessa Lawrence

**Affiliations:** a Department of Psychology, Institute of Psychiatry, Psychology and Neuroscience, King's College London, London, UK; b Department of Psychological Medicine, Institute of Psychiatry, Psychology and Neuroscience, King’s College London, London, UK; c Health Service and Population Research Department, Institute of Psychiatry, Psychology & Neuroscience, King's College London, London, UK

**Keywords:** chronic fatigue syndrome, cognitive behavioural therapy, recovery, qualitative interviews, reflexive thematic analysis, persistent physical symptoms

## Abstract

**Introduction:**

Chronic fatigue syndrome (CFS) is a chronic illness marked by severe, medically unexplained fatigue. Cognitive behavioural therapy (CBT) moderately improves fatigue and functioning. However, there is debate about recovery and how this should be operationalised. The impact of treatment on how recovery is viewed is also unclear. This study explored how people with CFS receiving CBT viewed recovery and whether these views differed at various stages of treatment.

**Methods:**

A total of 19 people with CFS receiving CBT were recruited from a specialist service in the UK. Purposive sampling was used to ensure a mix of age, gender, ethnicity and treatment stage. Semi-structured interviews were used to gather data and a reflexive thematic analysis was conducted.

**Results:**

The sample included 11 (57.9%) females and 8 (42.1%) males, with a mean age of 40 years old (range: 20−63). The mean duration of illness prior to treatment was 60 months (range: 12−142). The following four themes were identified in relation to recovery: (1) a personal process; (2) a reduction in symptoms; (3) a process of rebuilding, regaining, and retaining; (4) disrupting old ways of living. Theme Four was expressed mostly by those at later-treatment stages, suggesting that these emerged during treatment.

**Discussion:**

Recovery is a blend of ‘clinical recovery’, ‘personal recovery’ and ‘illness management’ models applied to other chronic conditions. Data suggests that concepts can change, and treatment may result in patients adopting views more in line with ‘personal recovery’ and ‘illness management’ models. These more flexible definitions, particularly those comprising changes to pre-illness beliefs and behaviours, new roles, acceptance and strategies to manage symptoms, corresponded with greater hope. Findings may help to inform realistic treatment expectations and contribute to more meaningful outcome measures.

## Introduction

Chronic fatigue syndrome (CFS) is a chronic illness marked by severe, disabling and medically unexplained fatigue, which is not alleviated by rest. Common symptoms include musculoskeletal pain, sleep difficulties and cognitive dysfunction (Collin et al., 2016). Some consider CFS and myalgic encephalomyelitis (ME) to be the same disorder, whereas others suggest ME is a different condition, with separate diagnostic criteria (Lim & Son, 2020). The current study uses the term CFS, rather than ME, as this has been operationalised in the literature. The prevalence of CFS in the UK population is estimated to be between 0.4% and 2.5% (Prins et al., 2006). If CFS is left untreated, only 7% of people fully recover (Cairns & Hotopf, 2005).

Cognitive Behavioural Therapy (CBT) is currently the only treatment recommended by The National Institute for Health and Care Excellence (NICE), which provides guidance on healthcare treatment in the UK (NICE guideline, 2021). This treatment is underpinned by a biopsychosocial, illness model of fear avoidance which proposes that initial fatigue symptomology is triggered by a viral infection or stressful event and that a subsequent combination of cognitive responses (e.g. fear of engaging in activity) and behavioural responses (e.g. activity avoidance) interact with physiological processes to perpetuate symptoms (Harvey & Wessely, 2009; Sharpe et al., 1996). The aim of treatment is to establish a regular rest and sleep pattern, gradually increase physical and mental activity, and to challenge unhelpful beliefs about symptoms and activity, including fears about symptoms or activity (White et al., 2011). Evidence suggests that CBT improves symptoms such as fatigue and physical functioning (Castell et al., 2011; Ingman et al., 2022; Larun et al., 2019; Maas genannt Bermpohl et al., 2024; Malouff et al., 2008; Marques et al., 2015; Price et al., 2008) with one trial (White et al., 2013) reporting a recovery rate of 22%. However, NICE guidelines (2021) suggest that CBT should be used to manage symptoms, improve functioning, and reduce the distress associated with having a chronic illness, rather than being seen as a ‘curative’ treatment. A lack of precise treatment recommendations may contribute to distrust and dissatisfaction amongst patient groups (Melby & Nair, 2024).

There remains debate about what constitutes recovery from CFS following treatments such as CBT. Whilst some argue that recovery is attainable (Knoop et al., 2007; Prins et al., 2001), others suggest that people improve by learning to cope with the symptoms of a chronic condition (Brown et al., 2011; Travers & Lawler, 2008). Some of this debate stems from a lack of clear pathophysiological mechanisms underlying the illness (Rivera et al., 2019), and from the fluctuating nature of symptoms, making recovery difficult to reliably ascertain. As such, different measures and definitions have been suggested and used, resulting in varied estimates (Adamowicz et al., 2014), whilst few clinical trials have reported recovery as an outcome (Marques et al., 2015).

Given the lack of consensus regarding how to operationalise recovery, there is a need to explore recovery from the patient’s perspective. This is because those with lived experience of CFS may be better placed to describe what recovery looks like, particularly as this may differ from clinicians or researchers (Knoop et al., 2007). In one qualitative study of 16 people who self-identified as recovered (Brown et al., 2017), recovery was seen as a self-driven endeavour involving a return to pre-illness functioning. However, participants continued to engage in ‘illness behaviour’, such as ‘boom and bust’ cycles of activity, as this allowed them to resume normal parts of their life, but also meant that they remained in a ‘liminal’ or ‘almost recovered’ state. In another smaller qualitative study (Devendorf et al., 2018), recovery entailed a return to old roles and identities with an absence of significant symptoms. Recovery for participants in both studies focused on regaining abilities but medical scepticism, and a perceived lack of treatment, contributed to uncertainty and a belief that ‘full’ recovery was unlikely.

Similar themes, including resumption of roles and functional improvement, were reported in a qualitative study of participants who had completed a brief, four session self-help graded exercise programme (Cheshire et al., 2021). Recovery for some participants in this study also involved working towards achievable goals, managing symptoms, and in some cases, altering pre-illness lifestyles or behaviours that were seen as undesirable or having contributed to the onset of illness. Similar to previous work, many viewed full recovery as ‘hoped for’, but unlikely, whilst some participants rejected the idea of recovery as a goal to be obtained if it disrupted their ability to live ‘in the present’.

In contrast to research focusing on people’s experiences during recovery, Bakken et al. (2023), conducted a narrative analysis on a group of fourteen people who considered themselves recovered. Recovery for these participants, who had suffered from very severe CFS, was precipitated by a shift in how they viewed their illness. For the central participant, who was used to illustrate a typical journey, this led to engaging in treatment focusing on reinterpreting symptoms as being controllable and not pre-determined by underlying pathophysiology. This was viewed as coinciding with changes at the biological level, including neurological and hormonal function. An alteration in beliefs about illness, involving ‘a long term, gradual learning process’, was therefore a key part of recovery for these participants, who no longer felt resigned to their symptoms.

In another narrative analysis of thirteen women who had experienced CFS as children, but had since recovered or improved, Krabbe et al. (2023) reported two ‘stories’ of recovery. The first involved building an understanding of bodily capacities and finding a balance between sleep, rest and activity. This included testing the body, taking risks, and learning from setbacks. The second story involved increasing activity, re-prioritising and paying attention to one’s own needs. The authors summarised recovery as a fragile, non-linear and self-driven process, which involved setbacks and ongoing adjustment. Recovery was therefore akin to being ‘reconnected to life’ but differed from their experience prior to falling ill as elements of both ‘illness’ and ‘wellness’ were present.

Extant literature suggests that recovery from CFS may therefore be viewed as including both a return to pre-illness functioning and an alteration of the pre-illness self, behaviours, identity or beliefs (Arroll & Howard, 2013; Travers & Lawler, 2008). Less is known about how treatment impacts these views of recovery, and the balance between recovering what has been lost and instigating change, and whether these views alter as treatment progresses. For example, some change, or aspirations for change, may be prompted by treatment, whilst some change may be an inevitable consequence of the illness experience, given the ‘biographical disruption’ and impact in multiple areas of life that this entails (Bury, 1982; Canguilhem, 2012; Charmaz, 1983). Indeed, without treatment, or medical, work and social networks lost through illness, changes to behaviour or beliefs may be necessary to survive, even if they perpetuate illness, or do not align with the values the individual holds. As previous authors have suggested, without treatment, those with CFS may feel the need to re-prioritise their lives, or even invent new, radically different selves (J. N. Clarke & James, 2003; Whitehead, 2006). Alternatively, treatment may engender change, or hope for change, that feels purposeful or that may otherwise feel unachievable (Bressan et al., 2017; McDermott et al., 2011). Further work is therefore needed to understand concepts of recovery in those with lived experience of CFS and to understand whether and how these views are influenced by treatment.

Exploring how patients view recovery in CFS within a CBT framework may have important clinical implications, and may help to inform realistic expectations for treatment (Adamowicz et al., 2014). The more accurately treatment aims are communicated to patients, the more this will increase treatment credibility, and the more likely positive change will occur (Di Blasi et al., 2001). Furthermore, a more comprehensive understanding of recovery might inform clinical outcomes measures that are more meaningful to patients*.*

### Aims

This study aimed to explore how people with CFS who were receiving CBT treatment viewed recovery and whether these views differed at various stages of treatment.

## Materials and methods

### Ethics

This study was approved by London - Chelsea Research Ethics Committee and the Health Research Authority (approval reference: 17/LO/2101). Prior to the interview, each participant was sent an information sheet detailing the purpose of the study. Author TI obtained written informed consent from all participants prior to enrolment in the study and informed participants that they could withdraw at any time and that their data would be anonymised.

### Study design

This was an interview-based, qualitative study using reflexive thematic analysis. This analysis was selected as it requires researchers to reflect on and acknowledge their subjective interpretations, important with complex illnesses such as CFS, where outcomes such as recovery are disputed. This analysis can also be used to organise and describe data in rich detail and highlight similarities and differences across data sets. The quality of the reflexive thematic analysis was guided by focusing on outlining the choice of methodology and a well-developed and justified analysis (Braun & Clarke, 2021)

### Sample

Participants were recruited from the Persistent Physical Symptoms unit, part of the South London and Maudsley NHS Trust. Treatment involved face-to-face cognitive behavioural therapy (CBT), a NICE-recommended intervention for CFS (NICE guideline, 2021) supported by evidence from UK-based trials (e.g. Adamson et al., 2020; Deale et al., 1997; White et al., 2011). Treatment was guided by a standardised manual (Burgess & Chalder, 2005) and was based on a model which assumes that certain triggers such as a virus and/or stress trigger symptoms of fatigue and subsequent coping behaviours perpetuate the symptoms. Treatment consisted of up to 15 fortnightly sessions, each 50−60 minutes in duration. Adaptations to treatment were agreed with patients, including written materials, notes and recordings of the sessions as well as shorter appointments, if needed (although most patients attending the Persistent Physical Symptoms unit tolerate hour-long appointments). A formulation of the individual's problems was discussed and agreed with each patient so the approach could be tailored to their needs. Each participant generated individualised goals at the beginning of therapy and in collaboration with their therapist. Therapists did not specifically inform patients whether full symptom remission was possible but instead encouraged them to focus on changing how they responded to symptoms with a view to eliciting helpful change. Whilst individual goals differed (e.g., increasing reading or socialising, tackling stress, changing sleep patterns), the cognitive and behavioural approaches underpinning treatment remained the same. Treatment was delivered by therapists experienced in the treatment of CFS, including two clinical psychologists and three CBT therapists, trained to post-graduate level, and accredited by either the Health Care Professionals Council (HCPC) or the British Association for Behavioural and Cognitive Psychotherapies (BABCP). All therapists received regular supervision.

Purposive sampling was used to ensure a mix of age, gender, ethnicity and treatment stages. Treatment stages were grouped as: ‘early-treatment’ (0−2 sessions), ‘mid-treatment’ (≥3 sessions), ‘late-treatment’ (follow-up sessions 3-, 6- and 12-months following treatment) and ‘post-treatment’ (completed all treatment). The boundary for ‘mid-treatment’ was chosen as initial therapeutic goals had been determined at this point of therapy, whilst the boundary for ‘late treatment’ was chosen as this is generally a period of consolidation and maintenance of learnt strategies.

Participants were recruited in two ways. First, therapists asked patients during routine out-patient appointments about whether they would like to participate in the research and then obtained permission for the research team to contact them. Second, participants were contacted by author (TI), who worked independently of the service, via the Biomedical Research Centre (BRC) Consent for Contact register, a database that includes patients who had already consented to be contacted for research. Of the 31 patients approached via either method, 19 (61.3%) consented. Participants were informed of the nature of the research and that their decision to take part would not affect their treatment. Participants received a summary of the findings at the end of the study.

### Inclusion/exclusion criteria

Inclusion criteria were: (1) a diagnosis of CFS according to Centre for Disease Control and Prevention criteria (Fukuda et al., 1994) or Oxford criteria (Sharpe et al., 1991) following a biopsychosocial assessment conducted by psychiatrists with expertise in CFS; (2) aged 18 or over; (3) those referred to, receiving or having recently completed CBT. Exclusion criteria were: (1) unable to speak English (2); a co-morbid diagnosis (e.g., bipolar disorder); (3) expression of suicidal intent. Researcher TI verified with therapists and clinic leads that participants met the above inclusion and exclusion criteria prior to approaching them for participation, and excluded any who did not.

### Data collection

The dataset was collected using one-off, semi-structured interviews conducted face-to-face or over the telephone by author (TI) or a research assistant (KH). Participants were given the choice of mode of interview. Interviews followed an interview guide initially created by the research team (the three authors) and then adapted and expanded on following consultation with CFS clinicians (including therapists, psychiatrists and occupational therapists) at the Persistent Physical Symptoms unit and a service user with CFS who had completed CBT at the Persistent Physical Symptoms unit. Unlike more positivist approaches, reflexive thematic analysis does not require that data be collected in the same way from all participants but instead treats the interview guide as a flexible tool that should evolve in response to the data and researcher’s developing insights. The researchers therefore took a recursive approach in which participants’ responses shaped the direction and content of the interviewer’s questions and initial findings from each interview were used to guide questions in subsequent interviews. Iteration allowed tailoring to context and participant experience and helped the researcher stay close to participants’ priorities and concerns, enhancing the depth and relevance of data (Clarke & Braun, 2013). Questions were therefore open-ended and not constrained by a predefined script. For example, interviewers became less inclined to ask whether participants had received information about recovery given that this did not elicit rich or relevant information about personal experiences of recovery and more likely to enquire about how concepts of recovery had changed - an early emerging theme. Nonetheless, key questions were asked of each participant, thus ensuring a baseline level of consistency between interviews and that interviews remained focused on the research question. These included: What does recovery from CFS mean to you? Where are you in the recovery process? What were your hopes and expectations of recovery? What factors are important to you in recovery? Why are they important? Has the concept of recovery changed as the illness has progressed? The full interview schedule, which includes both original questions devised by the research team, and questions generated as the study progressed, can be found in Appendix i.

Interviews were conducted in 2018 and lasted between 45 to 90 minutes. Interviews were recorded and transcribed by ‘Way with Words’ transcription service. Author TI listened to the interviews to check the accuracy of transcriptions and to increase familiarity with the data. Once transcribed, recordings were deleted, with all transcriptions stored on the Office 365 cloud, which was two-factor password protected.

### Analysis

The researchers adopted a critical realist ontological paradigm, which proposes that an external reality exists independently of those who observe it but is only known through perceptions and interpretations of individuals (Ritchie et al., 2014). An inductive, reflexive thematic analysis of the interviews, informed by the work of Braun and Clarke (2021), was conducted following the phases outlined below. The aim was to capture the participant’s perspectives and underlying conceptualisations at the latent level, without undue influence of existing theory. At the same time, reflexive thematic analysis emphasises the researcher’s subjectivity as analytic resource, and as such the researcher’s backgrounds, perspectives and assumptions were considered reflexively throughout the research process (Braun & Clarke, 2021; Braun & Clarke, 2022).

Two researchers (TI and KH), conducted the interviews. At the time of interviewing, TI was a 31-year-old, white British male in his final year of training as a clinical psychologist and KH, a 23-year-old white Canadian female who was completing a Master’s degree in Health Psychology. Both had previous research experience in CFS, but not clinical experience with this population. Three researchers (TI, KH and VL) were involved in the coding and qualitative analysis, whilst three researchers (TI, VL and TC) were involved in discussing the results and writing the current report. VL is a senior qualitative lecturer and has many years’ experience researching recovery in a variety of populations, but not in CFS, whilst TC has been conducting research and audits in relation to CFS for over 30 years. She has also assessed and treated many patients with CFS at various points in their journey.

Analysis was a recursive process allowing time for immersion and distancing. The first interview transcripts were read and re-read to increase familiarisation and look for patterns of meaning. Transcripts were then systematically coded according to the most basic element or observation considered meaningful (Boyatzis, 1998), for example, ‘symptoms’, ‘not working’ or ‘having routine’. TI coded all transcripts, KH coded three transcripts, and VL coded a further three. Coders compared and discussed codes to enhance reflexivity and increase the chances that interpretations remained close to the participants’ meaning. These discussions occasionally highlighted between-coder differences in interpretations. One example was differences in coding between personal views of recovery, the focus of the study, and ‘text book’ definitions of recovery, which was of less interest to the researchers. Following discussion, coders decided that this could be inferred by whether language was active or passive. Active language often emphasised the participant performing the action (e.g. “I want to be able to read for pleasure again”) whereas passive language was ‘through the eyes of others’ (e.g. “Most people I know, to them recovery means going back to being a normal who doesn't have fatigue”). Coders were then able to make a distinction between ‘recovery as a theoretical concept’, and the meaning of ‘recovery within individuals’ lives’, even if participants didn’t always make this explicit.

TI combined codes into themes, which were broader and more abstract than codes, and represented patterns of meaning underpinned by a shared concept or idea. As analysis progressed, themes were generated and reviewed and subsequent codes were incorporated into themes, if they fit. Themes were compared to coded extracts and the entire data set and if they did not fit, they were subsumed within other themes. Examples of subsumed themes include ‘feeling normal’, ‘independence’, ‘meaning and fulfilment’. Themes were then refined, defined and named (Braun & Clarke, 2006). During this process, TI remained vigilant as to how his beliefs might have influenced the research process, including his views that psychological therapies can be helpful for a range of physical and mental health problems, which may have potentially led to reporting treatment in a more positive light. Reflexivity was aided through diagramming, keeping a reflexive journal and TI discussing interpretations with other non-clinical raters (VL and KH).

Recruitment stopped when researchers felt they had achieved sufficient richness and depth of analysis (Malterud et al., 2016) and when each theme was refined across a diverse sample. Finally, the written report was produced, and excerpts used to exemplify each theme, and analysis discussed in relation to existing theory. Participants were assigned pseudonyms and any other identifiable information (e.g. age) was removed from patient extracts to maintain confidentiality. NVivo 11 software (for windows), which aids the organisation and visualisation of data, was used throughout analysis to generate codes and themes and to record prevalence of themes within and between participants.

## Results

### Participant demographics and treatment information

The sample included 19 participants, comprising 11 (57.9%) females and 8 (42.1%) males, with a mean age of 40 years old (range: 20−63). Of these, 10 (52.6%) participants were interviewed over the telephone and 9 (47.4%) face-to-face. Participants received a mean of 10 sessions (range: 0−18) of treatment, and none had dropped out at the time of interview. A total of 12 (63.2%) participants identified as white British, 3 (15.8%) as mixed/multiple ethnicities, 2 (10.5%) as British Asian, 1 (5.3%) as white other and 1 (5.3%) as black British. The mean duration of illness prior to treatment was 60 months (range: 12−142). Table I shows the mean age and duration of illness, and the proportion of female and Black and Minority Ethnic (BME) participants for each treatment stage.

**Table I.** Demographic and clinical characteristics for participants at each treatment stage.

	Early-treatment (*n* = 4)	Mid-treatment(*n* = 5)	Late-treatment (*n* = 5)	Post-treatment (*n* = 5)
Age (mean years)	41.3	45.4	36.0	40.8
Female (%)	75	60	80	20
BME (%)	50	60	20	20
Illness duration (mean months)	62.0	53.4	89.2	40.8

BME, Black and Minority Ethnic.

### Main findings

Four themes related to recovery were identified, which were as follows: *Theme One*: a personal process; *Theme Two*: a reduction in symptoms; *Theme Three:* a process of rebuilding, regaining and retaining; *Theme Four*: disrupting old ways of living. The following section describes each theme in more detail, including pseudonymised patient extracts.

#### Theme one: a personal process

Many participants, particularly those at the late- or post-treatment stages, referred to recovery as being individual, personal or idiosyncratic. Participants at the latter stages of treatment appeared more likely to adopt this perspective having been given more opportunity to reflect on personal and meaningful examples of improvement during treatment. One participant who expressed this view, also indicated that ‘generic’ definitions of recovery would not adequately capture or reflect the subtleties of her experience.

*“What is the clinical classification for recovery? If I was to do a questionnaire today then you might see me to be fully recovered. I suppose everyone’s definition of recovery is slightly different, isn’t it? If I was to see (name of therapist at clinic) and if we did a questionnaire, then she might say, well actually you’re X percent. I suppose everyone’s version of recovery is different.*”** (Eva, late-treatment)

Some of the most personal indicators of recovery related to visible manifestations of improvement, such as no longer requiring a wheelchair or walking stick. For one participant, who described himself as 80−95% recovered, recovery was being able to grow his hair again.

“*In a very visual way, my recovery is the fact I’ve now got my hair back…and they say, oh, is that the old Tony. Because when I was feeling so ill, I just got to the point… I had a bit of a Britney Spears moment and just shaved it off, because I was so tired and just the thought of having to style it or dry it or whatever, washing it, just having my arms above my head in the shower, was too tiring. I would just go to the barber every eight weeks and have it all shaved off. Not have to think about it ever. In some ways, that’s a very visual recovery, because I’m now back to how I used to look, a normal hairstyle.*”** (Tony, post-treatment)

Other participants, at various treatment-stages, referred to the individual nature of recovery more implicitly, suggesting that recovery depended on factors such as the severity of the illness, social support, financial circumstances and individual aims and motivation. These elements vary for each person meaning that there is unlikely to be one fixed version of recovery, as highlighted by one participant, post-treatment.

*“I guess it (recovery) depends on the sort of aims you set yourself. So, I suppose it depends on how bad and for how long you've had it.”* (Aaron, post-treatment)

Some participants felt that getting older resulted in a natural decline in energy levels, and they did not expect to be able to do the same things they could do when they were younger, irrespective of CFS. One participant was unsure of how much of an impact age would have on her recovery but found it helpful to compare herself to her peers who did not have CFS, suggesting that recovery varies according to diverse factors, including stage of life.

*“I realise I won’t ever be like I was before. However, I’m 40, I used to be 20 and I could do an awful lot more when I was 20. So, in my head I’m thinking, well, am I being unrealistic? Then I think, no, I can see people my own age are able to do a lot more than I can.”* (Nadia, early-treatment)

#### Theme two: a reduction in symptoms

All participants, at all stages of treatment, defined recovery in terms of a reduction in symptoms, such as unrefreshing sleep, less need for rest and pain after activity. Some participants spoke of a reduction in cognitive symptoms, such as difficulties concentrating or “brain fog” as being important. Most participants talked about the link between physical symptoms and secondary symptoms such as low mood and anxiety, and felt recovery would also involve an improvement in these.

***“**The physical tiredness. If I had that back, I think I’d just be happy again because still now I have a positive outlook in life. I’m a glass half full person and there’s still essentially a core of that there but it’s hard to feel that when you’re so tired.”* (Daniel, mid-treatment)

People’s experience of, and hope for, symptom reduction also contributed to this theme. Most at the late or post-treatment stage had experienced at least some improvement in symptoms, and had attributed this to CBT, a healthy lifestyle, Eastern medicine, or a mixture of these. Some participants, spanning various stages of treatment, defined “true” recovery as a complete remission of symptoms. There were a variety of beliefs about whether total symptom remission was possible. One participant, who was post-treatment, had experienced this, in addition to an increase in levels of activity.

***“**I think I would say that I’ve recovered because I don’t ever get symptoms anymore and I’m doing more than what I was doing before I was ill. So, I think yes, not having symptoms, once I stopped getting them was a real breakthrough and that's when I started to feel that I am actually better.”* (Aaron, post-treatment)

Others felt a total remission in symptoms was achievable, as they had witnessed others recover in this way, whilst some were much less hopeful, although there was no relationship between this belief and stage of treatment. Five participants, including one who was at the early-treatment stage, two at the mid-treatment stage, one at the follow-up stage and one who had completed all treatment, described their belief that a complete remission of symptoms was unlikely. This included one participant who defined recovery in terms of regaining previous energy levels.

*“Recovery for me is literally recovering that which has been lost. It’s normal ability levels and energy levels before this condition happened and being where it would be had it not been there…the only worry is that it won’t come for me. It’s not present. It’s the future. It’s a sort of conjectural state.”* (Daniel, mid-treatment)

Symptom reduction tended to become less important to participants as treatment progressed and other elements of recovery came to the fore, such as increased functioning, or symptom management (Themes Three and Four). One participant, who was at the post-treatment stage, said that he found it difficult to compare pre-illness levels of fatigue with his current levels of fatigue, as he was more physically active than before he became unwell, which he considered an acceptable and desirable goal. Other participants, including a small group who felt they had recovered, or had significantly improved, defined recovery as having fewer, or shorter relapses, suggesting that recovery and symptoms could co-exist. Another participant, at the late-treatment stage, explained that re-entering the work force had become her primary objective, and would help sustain the improvements in symptoms that she had made. She felt that home-based support or longer treatment may have helped with this, suggesting that future treatment could be adapted and refined.

*“I think there needs to be further support in place. That once you get to that point, for my symptoms to reduce a bit, there isn’t any support with the next steps to kind of help you with, right, let’s do some vocational things with you. You know, that seems to be missing, that once you get to that point of well, I’m feeling a bit better, now I need some support. I need all those vocational things. I need people's understanding to help me maybe tackle going back into the workforce.*”** (Alison, late-treatment)

#### Theme three: a process of rebuilding, regaining and retaining

Participants at all stages of treatment and self-identified stages of recovery likened recovery to a process or journey involving steps, stages, or a period of rebuilding. This often involved re-engaging in activities people had been doing before the onset of illness. One participant spoke about the impact of not being able to do the things that she could do previously, and why these were important for her sense of identity, purpose and place in society.

*“If you’re not able to kind of do all the things that you would normally have done with your time before you had chronic fatigue, you start to feel like you’re not important, that you don’t have a function in society. I think that’s part of recovery, is to feel that you actually have much more function in society so that your life can actually function better. And I think that will help your wellbeing because you’ll feel like you’ve got your purpose in life.”* (Alison, late-treatment)

Some participants, particularly at the early and mid-treatment stages, mentioned regaining independence. One participant, who experienced severe symptoms, spoke about being less reliant on others. For him, being able to wash without anxiety, or travelling on public transport, would represent important steps in recovery.

*“For me I think it would be not having to worry about standing up in the shower, because I’m worrying that I’ll fall down. You know, being able to go on a bus, for example. Just the little things that people, well I say normal people in inverted commas, take for granted.”* (Nathaniel, mid-treatment)

Participants’ goals for recovery evolved as treatment progressed. Most used returning to work or study in some capacity, without significantly worsening their symptoms, as an indication of recovery. Most also discussed the importance of social connection. Illness prevented many participants from being able to carry out activities that they used to enjoy or had been important to them, and recovery involved being able to do these again, often representing a state of “normality”. For example, one participant, who was at the beginning of treatment, wanted to be able to return to work, and do “everyday” things such as reading and domestic chores.

*“I want to be able to go back to work and there’s no way I can do that at the moment. I want to be able to read for pleasure again. It’s something I used to do an awful lot of and I can’t concentrate enough to do it. It’s very rarely now. I want to be able to be planning my next household task as I do one rather than thinking, well maybe in an hour’s time I’ll be able to do something. My garden’s a tip. I want to be able to do the gardening. These are all very everyday things which I used to take for granted and now I find impossible.*”** (Julia, early-treatment)

For another participant, who was in the follow-up stage of treatment, reading had also initially been difficult for her, but her concentration had improved to the extent that she was now able to do so every-day, and this was part of her recovery.

*“I’ve always been a really big reader and a writer, and for a long time my brain just didn’t work at all, and reading was this incredibly rare treat, but oh my God it was so wonderful when I could. Now I can read a bit every day, maybe not loads, and even if it’s just ten minutes, but I can still read every day, more-or-less.”* (Patricia, late-treatment)

Some participants, particularly those the beginning stages of treatment, felt rebuilding, regaining and retaining was contingent on a reduction in symptoms (Theme Two). This was illustrated by one participant, who was not hopeful that this was possible.

*“I’ve got quite a lot of things that I would like to be doing. I have interests in several things which it (CFS) has taken from me because I can’t physically follow these things up.”* (Daniel, mid-treatment)

On the other hand, another participant who had completed treatment, and who described himself as fully recovered, explained that it had been important to see improvement in functioning, even in the presence of symptoms. In his case, he seemed to benefit from the goal-focused nature of CBT which confirmed his view of recovery as a gradual increase in functioning.

*“So, I think for me, I'm quite goal orientated, so a lot of stuff I do is about reaching something. So, what we did is we worked on a weekly plan of how much and what I was going to do in that particular week ….It was like right, this week I'm going to try to walk for ten minutes, read for half an hour, and it was all a building process, which I found quite good for me. I found that I need that sort of structure and that sort of progression, so I could see it was getting better.”* (Aaron, post-treatment)

As treatment progressed, recovery was more likely to be viewed as a complex, non-linear process involving setbacks, without a specific ‘end-point’. One participant, who said that she did not feel fully recovered, but was improved, felt that setbacks were unpredictable and continued to experience them even after treatment had finished. Recovery was therefore a constant work in progress, with ‘good weeks’ involving doing more of what was important to her.

*“And week to week, month to month, it’s different again. So, you might have a good week but next week it could be downhill again….So to me, there is really no cut-off point when I feel like I’ve recovered because something happens. It’s not really as simple as that.”* (Hina, post-treatment)

Another participant, who had had been ill for 10 years, and had been bedridden, compared his recovery to seasonal changes, which had become less severe as he improved. In his case, rebuilding was a cyclical process, with periods of feeling unwell and reduced activity (the “winter”) eventually making way for periods of increased energy and activity (the “summer”). This highlights the importance of language used in treatment, and the impact this has on people’s interpretation of symptoms, as this metaphor was more motivating than language implying low points and high points in activity and health.

*“I feel like I go through periods of kind of my body is going through like a bit of winter or, you know, and sometimes I feel like, yes, it’s the kind of it’s the real like summer moment. It’s kind of a funny metaphor but like I feel like you go through cycles and those cycles have become less intense. And I feel kind of, yes, psychologically that the, kind of, up and down peaks and troughs is not a helpful metaphor because you don’t like to feel like you’re going downhill again and then you have to climb up again.”* (Isaac, late-treatment)

#### Theme four: disrupting old ways of living

Some participants explained that recovery involved resuming aspects of their lives before the onset of CFS (Theme Three) whilst simultaneously making changes to those aspects. This often related to changes in the quantity or intensity of old roles, such as work, as well as discovering new roles or skills. For some, recovery was also about accepting symptoms and developing and maintaining methods for managing these. Theme Four was articulated mostly by those who were at the mid-, late- or post-treatment stages, except for one participant at the early stages of treatment who had previously had CBT for depression, suggesting that this aspect of recovery emerged during treatment.

Two participants who had completed most, or all, of their treatment, spoke of wanting to make changes to the lifestyle they had before they became unwell. One participant, who was severely affected by her symptoms, felt that a drive to always do more, coupled with her belief that she was never doing enough, had contributed to the onset of CFS, and she was keen to change this.

*“There’s a part of me that thinks I can do everything I used to do and did before, but I also realise that I don’t want to go back to how I was before and I did way too much, which was partly why I’m in this position. I didn’t have this gauge that I guess a lot of…you know like some people keep drinking and they don’t have that thing in them that tells them when they’re drunk and so they just keep on drinking and drinking? But I was like that in terms of activity. So, in my head nothing was ever enough; I was never doing enough, I always needed to do more, and I just didn’t have that gauge. It was like a broken compass that was saying this is too much.*”** (Patricia, late-treatment)

Another participant felt that making changes to his “previous” life, including having greater variety and balance, was integral to recovery.

*“Recovery means not only feeling better, but also having a better-balanced life and having changed my life. The two are interlinked, inextricably. I don’t think I could have had my life as it was and been ill and gone back to my old life. I had to change it as well. That change is definitely part of the recovery.”* (Tony, post-treatment)

In addition to emphasising change, both of these participants had endorsed other themes of recovery (e.g., as a personal process involving a reduction in symptoms as well as regaining old roles). This implies a multi-dimensional, flexible conceptualisation of recovery, which appeared to be associated with greater hope. Both participants were also at the latter treatment stages which suggests that treatment may help to elicit these more flexible views. Conversely, participants who considered recovery only in terms of a return to previous levels of functioning, tended to be less hopeful for recovery.

One participant, who was mid-treatment, and was severely affected by his symptoms, spoke of his desire to help other people with CFS. He was also keen to challenge the narrative that CFS was “all in the head”. The ability to enact these new roles, which were not considered part of his pre-illness identity, would constitute his recovery.

*“Recovery for me, or complete recovery for me, would be being able to do something in some small way to help someone else not to have to go through what I’ve gone through…which is why I do tend to… I was very thankful when you asked me to be involved in this, because I wouldn’t really want this for anyone else.”* (Nathaniel, mid-treatment)

Some participants in the mid-, late- and post-treatment stages also described recovery as having “strategies” or “tools” to draw on in case they needed to manage their symptoms. This included not over-extending themselves, ensuring adequate sleep, reducing stress, being more assertive or challenging unhelpful thoughts. One participant expected to continue utilising these strategies for the rest of her life.

*“I think this is now a lifelong way of living really, and I feel like if I was to suddenly stop doing all of the things that I’ve learnt to do and all of the strategies; I feel like I could go back, maybe not right to the start, but I could certainly relapse again and find myself in the position I was in a couple of years ago, which was basically not really able to leave the house.”* (Eva, late-treatment)

Being more ‘in tune’ with body and mind was another common concept. For example, when asked about her hopes for recovery, one participant said that she wanted to have greater awareness of her limitations so that she could maintain a better life balance, which she felt she had achieved. One participant, who described himself as fully recovered, felt confident that he had the tools to manage symptoms if they did return, and attributed this to CBT.:

*“I think just having the awareness of your body. Just knowing that there are limits of what you can do, but I think I've become aware of it and having had all the treatment and discussions with (therapist name) and stuff….If it did come to the stage where I was going back and feeling tired, I’ve got ways of dealing with it and I know what to expect and how to alleviate it, I think.”* (Aaron, post-treatment)

Managing symptoms in this way, also meant accepting that symptoms may be present*.* For one participant, who felt he was close to recovered, accepting the presence of his symptoms was a necessary part of recovery and had provided him with greater focus in life. However, he had initially considered recovery as an absolute return to previous levels of functioning, reinforcing the notion that definitions of recovery can change:

***“**When I first got ill, I thought, I just want to be like how I was. I don’t want to feel tired or ill anymore. But now I know I can still do things whilst not feeling 100%. And actually, it’s made me a bit more, not dynamic or focused, but slightly more… Yes, slightly more focused to get more out of life. In some ways, the illness had a good effect. A recovery has actually made my life better in some ways.”* (Tony, post-treatment)

For another participant who, at mid-treatment, who did not feel that his symptoms had been alleviated, accepting the limitations imposed by his illness also represented a change, and a helpful cognitive shift, which he believed could help him to regain some of what he had lost. Acceptance did not represent recovery, as he had not regained anything but appeared to be a necessary first step before any recovery or “salvaging” could occur.

*“For me it’s about acceptance and working with what I’ve got. ….So that I suppose, acceptance, working with it, would be a mental shift…. Acceptance of the condition as it is. I’m not recovering anything. At the moment it’s still a loss for me. But what there is acceptance of my limitations and some adjustment to try and salvage something.”* (Daniel, mid-treatment)

For both of these participants, acceptance was a psychological adjustment which helped them acknowledge and adapt to the reality of their symptoms. This adjustment was seen as either an important part of the process of recovery or helped lay the foundation for future potential recovery. This differed from pre-treatment views, where symptoms were seen as intolerable, or were denied or avoided, leading to unrealistic attempts at functioning, and unintentionally contributing to greater mental and physical anguish. Similarly, another participant, Patricia, who reported severe fatigue, impairment, and disability, demonstrated a significant mental shift, appearing more willing to accept and tolerate uncertainty about the future. Interestingly, this view co-existed with greater hope and putting maximum effort into the recovery process, suggesting a more flexible concept of illness and recovery. All three of these participants appeared to demonstrate how acceptance represented a new cognitive skill that helped them move towards recovery, and potentially be more open to life’s struggles.

*“I had this massive mental turnaround where I just thought, you may never ever get better; you have no idea what’s going to happen and you may never recover and it may never get better than this. However, if you don’t try, it’s definitely not going to happen. If you do try, it still might not happen, but at least you’ll have tried and you’ve done something about it. And I just had this sudden thing of, you’ve got to put 100%, you’ve just got to throw your entire self into this and just completely go for it, 100%, and just throw everything at it.”* (Patricia, late-treatment)

## Discussion

### Main findings

This study investigated concepts of recovery in CFS patients at various stages of CBT treatment. In summary, most participants considered recovery to be a reduction in symptoms and a return to previous roles. This is similar to ‘clinical recovery’ models, with a focus on ‘restoration’, which is favoured by some clinicians (Devendorf et al., 2017; Slade, 2009). This view of recovery tended to become less prominent as treatment progressed, and other elements of recovery came into focus. This included monitoring and managing symptoms. Previous work has described this as ‘illness management’ which is an aim in chronic illnesses such as hypertension and diabetes (Gingerich & Mueser, 2005). Others, particularly at later stages of treatment, referred to recovery as an individual process, or journey, involving change and acquiring new ways of living. This shares elements of the ‘personal recovery’ model often used with mental health settings and is favoured by the disability rights movement and cancer survivors. These findings will now be discussed in more detail.

The finding that restoration, or returning to some level of pre-morbid baseline functioning, is a key aspect of recovery is reflected in previous qualitative literature. These include recovery being seen as a reduction in symptoms and/or a resumption of pre-illness roles in work, family or social domains (Brown et al., 2017; Devendorf et al., 2018). This is often framed by people in both the current study and previous work as ‘returning to normal’ (Joachim & Acron, 2000). Whilst recovery became more than just a process of restoration for many participants in the current study, particularly as treatment progressed, most participants thought there was some chance of this type of clinical improvement. This contrasts with previous research that found that most CFS participants had low expectations for a ‘clinical’ recovery (e.g., a remission of symptoms). Indeed, whilst previous literature (Brown et al., 2017) reported that recovered participants had not undergone a clear cut clinical improvement, the findings of our study suggest this is possible, as demonstrated by one participant who reported complete remission of symptoms. These differences may be related to lack of access to treatment (e.g. Brown et al., 2017; Devendorf et al., 2018) or the type or intensity of treatment. For example, Cheshire et al. (2021), investigated a guided self-help graded exercise programme - a less intensive treatment which lacked the cognitive components of CBT. Access to a more comprehensive course of CBT and the support of healthcare professionals, as well as collaborative goal setting between therapist and patient, may have positively impacted on people’s hope for and experience of recovery. This is reflected in the more modest goals identified at the early- and mid- treatment stages of treatment, such as self-care, compared to more ambitious goals at the later stages of treatment, such as returning to work. Nonetheless, a minority in our study desired a full ‘clinical’ recovery, which they felt was unlikely or unachievable. This therefore may not be a realistic goal for all undergoing treatment, particularly if this is viewed as the sole objective for recovery.

As treatment progressed, however, other objectives, over and above ‘clinical’ recovery, tended to emerge. This suggests that, as well as CBT helping some participants ‘move towards’ recovery, treatment also impacted people’s views of recovery. For example, treatment elicited more nuanced views of symptoms and functioning, akin to how people conceptualise these and cope with chronic illness. Views on coping varied and were inevitably influenced by CBT, and the language used in this treatment. For example, participants described “tools” or “strategies” to manage symptoms, or wanting to be more “in tune” with mind and body, although these terms have been described elsewhere (Brown et al., 2017), and are therefore not unique to CBT. Some participants in our study, as well as in previous work (Bakken et al., 2023), coped by re-interpreting symptoms as not to be “feared” or to result in avoidance. For one participant, who had completed treatment, and described concepts that corresponded with an ‘illness management’ view of recovery, accepting their symptoms and continuing to do meaningful activities even if not feeling “100%” was key. Indeed, previous work on people with chronic illness has shown that they may regain functioning, such as returning to work, even if symptoms persist, as they consider this a key part of coping (Bury, 1982). For another participant, who was mid-treatment, and appeared to subscribe to a ‘clinical recovery’ model, accepting their symptoms did not represent recovery per se, but was a necessary first step towards potentially regaining elements of life prior to being unwell. Participants such as these felt that recovery involved acceptance of symptoms, which in turn helped them face the challenges of illness. This is consistent with previous work showing that acceptance of symptoms in CFS is important (Travers & Lawler, 2008; Wilson et al., 2011) and can be facilitated by CBT (Brooks et al., 2011). This may also be why ‘third wave’ cognitive behavioural therapies, such as Acceptance and Commitment Therapy, which emphasise accepting rather than eliminating symptoms, and living according to important values, show promise in the treatment of CFS (Clery et al., 2021; Jonsjö et al., 2019).

Some individuals, particularly at the later stages of treatment, felt that recovery involved continued use of the strategies they employed to manage symptoms, even in the case of participants who considered themselves recovered. This suggests that people saw recovery as involving the potential for productive and fulfilling lives despite the possibility of symptoms or their illness returning, and the need to adjust when this happens. This aligns with both the ‘illness management’ and ‘personal recovery’ models. The continuing need to manage symptoms, without a specific end point, may be why previous CFS literature has highlighted that people struggle to identity as ‘recovered’, even if they have resumed ‘normal’ activities. As Krabbe et al. (2023) highlighted, both ‘illness’ and ‘wellness’ may be present if people are continually adjusting to symptoms. However, treatments such as CBT may provide hope that recovery can entail a workable balance between having symptoms and utilising methods for coping with these symptoms, or keeping them at bay. This was demonstrated in the case of one participant who had completed treatment, and was still experiencing significant symptoms, but who was hopeful that full recovery was possible with the necessary tools. This viewpoint was motivating for this participant, and coincided with elements of the ‘fighting spirit’ adopted by some people with cancer, in which people view illness as a challenge (Watson et al., 1988). This is in stark comparison to some participants in Devendorf et al. (2018), who did not have access to treatment, and felt that ‘fighting’ the illness was futile and would lead to stress and disappointment. This highlights how access to treatment may elicit greater hope for recovery.

Other concepts of recovery that were more prevalent in the mid-to-later stages of treatment, rather than early stages, included viewing recovery as a personal process. This may be because therapy provides the opportunity to reflect on the nuances of recovery, and as treatment progresses, the focus shifts to more personalised goals. Time also appears to contribute to viewing recovery as a personal endeavour, as people naturally adjust to illness, and grow older, and their values, goals and expectations change (Bakken et al., 2023; Brown et al., 2017; Groven & Dahl-Michelsen, 2022). The idea that recovery is a relative concept is shared by clinicians who suggest that expectations for functioning should be based on healthy-age peers (Devendorf et al., 2019; Griffiths et al., 2015). However, participants in our study emphasised that purely function- or symptom-based definitions would not capture their unique experiences. This mirrors research into stroke, in which measures of recovery focusing on function and capacity may be too simplistic (Dowswell et al., 2000). This suggests that a move towards more holistic measures incorporating physical, emotional and social domains and person-centred facets of recovery (Hommel et al., 2016) may be more appropriate.

Other shifts in how people viewed recovery which appeared to be precipitated by CBT included alterations to some pre-illness attitudes and behaviours, and acquiring new roles, again reminiscent of the ‘personal recovery’ model of recovery. Some of the changes participants identified and worked towards in their recovery included rejecting elements of pre-illness lifestyle, such as excessive activity and unrelenting standards. Whitehead (2006) reported similar findings in their work in untreated individuals who, over time, made self-imposed changes to pre-illness patterns they felt had been detrimental to their health. However, findings from this study differ from the drastic shift described by participants in Clarke and James (2003), who sought a separation from their former lives. It may be that treatments such as CBT are helpful in adapting ‘action-proneness’ and finding a more healthy life balance that precludes a need for completely rejecting previous roles (van Houdenhove et al., 2006; Ware, 1993). This was also described by participants treated with graded exercise therapy, in Cheshire et al. (2021), who defined recovery as an altered lifestyle, taking into account those factors that led to their becoming ill in the first place.

In addition to shifts in pre-illness lifestyle, some participants viewed recovery as incorporating aspirations for ‘new roles’, including helping others through illness. This is commensurate with the concept of ‘reconstruction’ described by Travers and Lawler (2008), in which people attempt to find new areas of fulfilment, the ‘quest narrative’ described by Whitehead (2006), which involves an altered perspective on life, and ‘post-traumatic growth’ outlined by Arroll and Howard (2013), in which the distress of illness can give rise to positive growth. The idea that positive adjustment can follow a life crisis has been described in other chronic conditions, such as cancer (Brennan, 2001). This type of adjustment may enable people to view personal or physical trauma as an opportunity to focus on the important, meaningful aspects of life, which can constitute another key part of recovery, and which our findings suggest may be more likely to emerge during treatment.

Taken together, findings from the current study indicate that CBT treatment may elicit more flexible views of recovery that share similarities with the ‘illness management’ and ‘personal recovery’ models. These definitions of recovery include a rejection of previously unhelpful ways of living, adopting new roles and identities, acceptance and tools to manage symptoms. Flexible views of recovery and illness such as these may give rise to increased hope and better outcomes (Adamowicz et al., 2014; Arroll & Howard, 2013). Conversely, those participants who held predominantly ‘clinical’ definitions of recovery, involving total symptom remission and restoring what is lost without change, generally had lower hope for recovery. The ‘clinical’ model of recovery was held by participants at various stages of treatment, including one participant at the end of treatment who did not feel treatment had been long enough or had provided her with enough home-based support. This latter participant expressed less hope for recovery and may represent the significant minority of people who feel they do not improve following current forms of CBT.

### Clinical implications

These findings will enable clinicians to more effectively communicate concepts of recovery to patients at the beginning of treatment which may make change more likely to occur (Di Blasi et al., 2001). These findings will also help to align treatment goals and outcomes, thus improving treatment credibility amongst patients. Clinicians may also wish to explore elements of ‘illness management’ and ‘personal recovery’ whilst working with harder-to-treat patients. This might include focusing on changes to pre-illness lifestyle, exploring new roles and identities, acceptance, and strategies to manage symptoms. These patients may also benefit from more holistic, home-based support, including help re-entering the workforce, or obtaining financial benefits, particularly for those who are isolated (Drachler et al., 2009). Finally, whilst some clinicians privilege ‘clinical recovery’ (i.e., complete symptom remission) over ‘illness management’ (i.e., coping or managing symptoms), not everyone with CFS subscribes to the former, and some may feel recovered despite experiencing, or the possibility of experiencing, symptoms. Thus, conventional symptom- or function-based outcome measures should ideally be supplemented by measures capturing coping and quality of life pertaining to physical, emotional and social domains.

### Limitations

There were some limitations of this study. Recruiting participants from secondary care may have limited the range of experiences explored. Those who were housebound were less likely to access community services, therefore findings from this study may not have captured views of the most severely ill. As always in participant recruitment, there may have been a bias among those who agreed to participant, who may have been different in some way from those that did not. For example, participants who agreed may have had stronger opinions, positive or negative, about therapy and recovery. With regard to data collection, the use of a recursive interview approach meant that interviews were unstandardised, thereby increasing the potential for researcher bias. However, this method is consistent with the epistemological foundations of reflexive thematic analysis, which emphasise the co-construction of meaning rather than standardisation or neutrality. The recursive approach necessitates a high degree of researcher reflexivity and systematic documentation to ensure that themes remain firmly grounded in the data. A further limitation is that this study explored how people with CFS viewed recovery through the lens of CBT treatment, and therefore may have been influenced by the clinicians involved in their care, and their expectations for recovery. However, as treatment progresses, the patient’s conception of recovery is more likely to be grounded in their own experiential changes rather than in the beliefs and expectations of the therapist. This is consistent with evidence indicating that therapist-related variance does not significantly influence clinical outcomes in CFS patients receiving CBT (Cella et al., 2011). Nonetheless, patients receiving a different treatment would likely have different expectations and views on recovery. A final limitation is that it is now more common in qualitative research for people with lived experience to be involved in analysing data or checking preliminary themes. This approach may help to ensure that service users’ voices are heard, and lead to findings that may otherwise have been missed. This was not feasible within the time constraints of the study. It is therefore important to acknowledge that a different research team may have produced a different analysis and set of findings, a variability inherent to qualitative research.

### Future research

Future studies may wish to explore whether focusing on ‘illness management’ and ‘personal recovery’ during treatment may improve outcomes, particularly in harder-to-treat patients. A feasibility study may also be useful to explore the viability of a ‘personal recovery’ or ‘illness-management’ based measure for CFS, given that these are often used to measure outcome in chronic physical and mental health conditions (Schmitt et al., 2013; Simoni et al., 2006; Young & Bullock, 2005).

## Conclusions

Recovery in CFS is a personal process involving symptom reduction, resuming old roles, disrupting unhelpful ways of living and having strategies to manage setbacks. Therefore, recovery blends ‘clinical recovery’, ‘illness management’, and ‘personal recovery’ models often applied to other physical and mental health conditions. However, concepts of recovery can change, and treatment may result in patients adopting views more in line with ‘personal recovery’ and ‘illness management’ models of recovery. Generally, these more flexible definitions of recovery, particularly those comprising changes to pre-illness beliefs and behaviours, new roles, acceptance and strategies to manage symptoms, were related to greater hope. These findings will enable clinicians to more effectively communicate concepts of recovery to patients, potentially improving credibility and outcomes. Future studies may investigate whether harder-to-treat patients benefit from treatments that emphasise flexible elements of recovery and explore measures to capture these outcomes more effectively.

## Data Availability

The data that support the findings of this study are available on request for the corresponding author (TI). The data are not publicly available due to their containing information that could compromise the privacy of research participants.
